# TLR3, TLR7, and TLR8 genes expression datasets in COVID-19 patients: Influences of the disease severity and gender

**DOI:** 10.1016/j.dib.2024.110498

**Published:** 2024-05-03

**Authors:** Nasir Arefinia, Parsa Banafi, Mohammad Amin Zarezadeh, Hawra Shah Mousawi, Ramin Yaghobi, Mehrdad Farokhnia, Jamal Sarvari

**Affiliations:** a Department of Bacteriology and Virology, School of Medicine, Shiraz University of Medical Sciences, Shiraz, Iran; b Bio Environmental Health Hazards Research Center, Jiroft University of Medical Sciences, Jiroft, Iran; c Transplant Research Center, Shiraz University of Medical Sciences, Shiraz, Iran; d Department of Internal Medicine, Faculty of Medicine, Afzalipour Hospital, Kerman University of Medical School, Kerman, Iran; e Gastroenterohepatology Research Center, Shiraz University of Medical Sciences, Shiraz, Iran

**Keywords:** Toll-like receptors 3 (TLR3), TLR7, TLR8, Severe acute respiratory syndrome coronavirus 2, Coronavirus disease 2019

## Abstract

The prognosis of COVID-19 could influence by innate immune sensors such as toll-like receptors (TLRs). The purpose of this data was to investigate TLR3, 7, and 8 expression levels in COVID-19 patients and their relationship to outcome of disease.

75 confirm COVID-19 were included sequentially and separated into three groups: mild, severe, and critical. Peripheral blood mononuclear cells were isolated from the whole blood, and RNA was then extracted. The qRT-PCR technique was used to examine the expression of TLR3, TLR7, and TLR8 genes.

The patients average ages were 52.69 ± 1.9 and 13 of the 25 individuals in each group were male. TLR3 (*p* < 0.001), TLR7 (*p* < 0.001), and TLR8 (*p* < 0.001) expression levels were considerably greater in COVID-19 patients compared to the control group. The findings also showed that individuals with critical and severe COVID-19 disease had significantly greater TLR7 and TLR8 gene expression levels than patients in mild stage of disease (*p* < 0.05). The data showed a significant difference (*p* = 0.01) in the TLR3 transcript levels between critical and mild COVID-19 patients. Furthermore, male severe (*p* = 0.02) and critical (p = 0.008) patients had significantly higher TLR8 expression levels than female patients in terms of gender. TLR3 (*p* = 0.2) and TLR7 (*p* = 0.08) transcripts were more elevated in males than females, but not significantly.

Specifications Table

**Table:** 

Subject	Immunology
Specific subject area	Expression of Toll-like receptors in COVID-19 patients
Data format	Raw, Analysed
Type of data	Table, Figure
Data collection	Blood samples were collected from 75 COVID-19 patients and 25 healthy controls. PBMCs were isolated using density gradient centrifugation. RNA was extracted and TLR gene expression was measured using qRT-PCR. qRT-PCR amplifications with 96 to 106 % efficiencies were set up in triplicate with a 10 µL volume containing 5 µL SYBR green master mix, 1 µL of the corresponding cDNA, and 10 pMol primers. Amplification efficiencies were calculated and included in data normalization. Analysis of the melt curve confirmed the specificity of qRT-PCR. The β-actin gene was also used as an internal control and the levels of the TLR3, TLR7, and TLR8 expression were calculated by Pfaffl formula.
Data source location	Shiraz University of Medical Sciences, Shiraz, Iran
Data accessibility	Repository name: Mendeley Data Direct URL to data: https://data.mendeley.com/datasets/yxj2mtmthd/1
Related research article	-

## 1 Value of the Data

• This dataset provides novel insights into TLR gene expression patterns in COVID-19 patients across disease severity levels. The data show increased expression of TLR3, TLR7, and TLR8 correlates with more critical illness, suggesting these genes may be biomarkers for prognosis.
• These are the first data demonstrating TLR8 expression differs significantly between male and female COVID-19 patients, with higher expression in critically ill males. This implies TLR8 could underlie gender differences in COVID-19 mortality and severity.
• The dataset enables other researchers to further investigate connections between TLR activation and aberrant inflammatory responses in severe COVID-19. Dysregulated cytokine release downstream of TLRs likely contributes to adverse outcomes.
• Other groups can build on these findings to assess whether TLR antagonists have therapeutic potential for mitigating harmful inflammation in critical COVID-19 cases. The data provide a rationale for targeting TLRs pharmacologically.
• This dataset can be integrated with other COVID-19 gene expression data to construct more comprehensive models of SARS-CoV-2 immunopathogenesis. Elucidating mechanisms leading to severe disease remains a priority.

## 2 Background

In 2020, the severe acute respiratory syndrome coronavirus 2 (SARS-CoV-2) spread globally, infecting many people and causing a massive health disaster. SARS-CoV-2 causes the coronavirus disease 2019 (COVID-19) and in some cases it can cause pneumonia and acute respiratory distress syndrome (ARDS) syndrome, causing involvement of organs such as heart, kidney, and pancreas and even death [1].

Numerous concerns regarding the pathophysiology of this disease remain unsolved. The pathogenesis and severity of viral diseases can be influenced by viral and host factors. Among viral factors, viral proteins and mutations in the viral genes are the most important influencers. Among host factors, age, gender, underlying chronic diseases, previous immunity, and genetic background play important roles [2]. The immune system stands out among these as possibly having a significant impact on COVID-19 progression. Mammals fight diseases with two different types of immunity: innate immunity and adaptive immunity. As a pathogenic sensor, intrinsic immunity aids in the eradication of infections and the development of compatible immunity [3]. Pattern recognition receptors (PRRs) play a key role in these tasks. Toll-like receptors (TLRs), a class of transmembrane proteins, are PRRs that have a potent potential to activate antigen-presenting cells (APCs) and strengthen the immune system. In humans, there are 10 members of the TLR family (TLRs 1-10) [4].

The immunopathogenesis of SARS-CoV-2 infection affects the immune system may be significantly impacted by the activation of different TLR pathways. This activation leads to the production of proinflammatory cytokines, including interleukin 1 (IL-1), IL-6, tumor necrosis factor (TNF), and interferon type I (IFN-I) [5]. Research has shown that COVID-19 patients have higher levels of TLR3, TLR7, and TLR8 mRNA expression in their nasopharyngeal epithelial cells compared to healthy individuals [6]. Additionally, Egyptian COVID-19 patients have been found to have higher levels of TLR2 and TLR4 expression than healthy volunteer groups [7]. TLR3 is one of the most significant TLRs since it recognizes dsRNA and can promote the production of type I interferons via IRF3 [8]. High-level expression of TLR3 has been shown in nasopharyngeal epithelial cells of COVID-19 patients compared with the control group [9]. According to a study, TLR3 levels rise in COVID-19 patients within the first 24 h of the illness, which triggers the the production of inflammatory cytokines such as IL-1, IL-4, and IL-6 [10]. Also, another study conducted on influenza infection showed that TLR3-/- fibroblast cells did not have the ability to produce beta and gamma interferons [11].

Among other members of the TLR family, TLR7 shows a role in inducing inflammation and producing type I interferon by identifying GU-rich motifs in single-stranded RNAs of foreign microorganisms [12]. According to scientific experts, TLR7 is crucial in triggering effective immune system responses to SARS-CoV-2 infection. This is supported by the fact that individuals who lack TLR7 are more likely to develop a severe form of COVID-19 [13]. Studies have shown that the transcriptional levels of TLR7 are higher in the nasopharyngeal epithelial cells of COVID-19 patients than to healthy individuals [9]. In patients with HCV infection, the expression of the TLR7 gene was found to be higher in non-responders compared to responders [14]. Also, deficiency in the function of TLR7 caused disruption in the production of specific antibodies against the influenza virus [14].

TLR8 is another important TLR that can recognize GU-rich single-stranded RNA and oligonucleotides in the endosome. Research has shown that COVID-19 patients have higher levels of TLR8 expression in their nasopharyngeal epithelial cells than those who in healthy group [9]. In HBV and vaccinia virus infections, it has been reported that TLR8, by inducing IFN-alpha and beta, plays a crucial role in managing the infection. Also, in influenza A (H3N2) and HIV1 infections, TLR8 induces inflammation to control infection by inducing the expression of IL-6 and 8 [[15], [16], [17]].

Studies have shown heightened expression of several Toll-like receptors, such as TLR1, TLR2, TLR4, TLR6, and TLR9, in nasopharyngeal epithelial cells sampled from patients with severe COVID-19 [6,18].

Emerging evidence indicates Toll-like receptors play a key role in COVID-19 pathogenesis. According to recent findings, the Omicron variant's reduced virulence may stem from decreased TLR stimulation. This is proposed to minimize NF-κB activation, furin production, and viral replication in lung tissues compared to other variants [19]. Therefore, scientists have suggested that TLRs agonists and antagonists might be useful targets for COVID-19 treatment [5]. Therefore, the purpose of this study was to examine the expression levels of TLR3, TLR7, and TLR8 in COVID-19 patients and their relationship to the severity and outcome of the disease.

## 3 Data Description

This case control study involved a total of 100 subjects divided into 4 groups of 25 individuals each. The groups consisted of 25 patients with mild COVID-19, 25 with severe COVID-19, 25 with critical COVID-19, and 25 healthy volunteers. Within each patient group, there were 13 males and 12 females. The average ages (±SEM) of the mild, severe, critical patients and healthy volunteers were shown in Table 1. In addition, about all data we encourage researchers to see https://data.mendeley.com/datasets/yxj2mtmthd/1.

**Table 1:** The demographic features of the three study groups and the healthy volunteer individuals.

		Mean	SEM	IQR	P-Value
Age	Healthy	49.12	2.7	25.4	0.253
	patients	52.69	1.9	22.6	
	Group	Mean	SEM	IQR	P-Value
Age	Mild	52.840	2.70	22.5	0.627
	Severe	51.080	2.40	25	
	Critical	54.280	2.44	18	

**Fig. 1:**
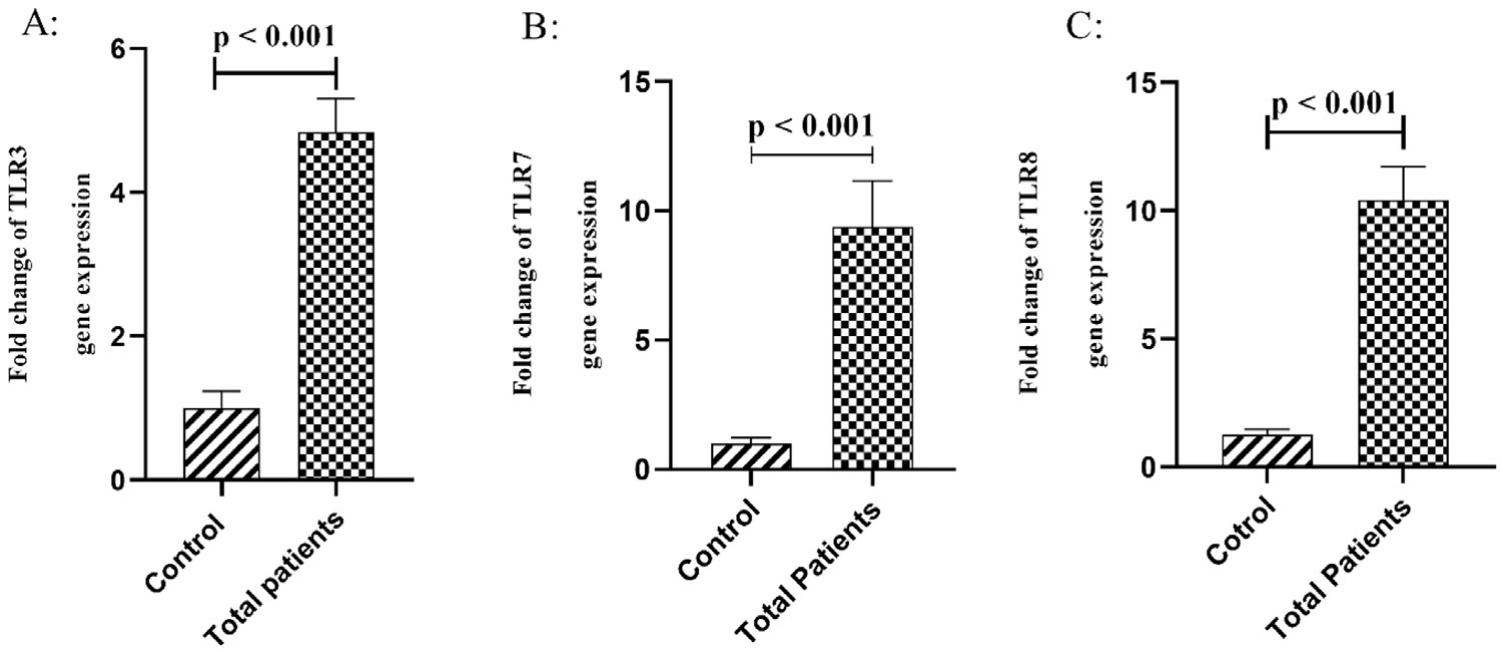
TLR3 (A), TLR7 (B), and TLR8 (C) gene expression in peripheral blood samples from COVID-19 patients and the control group. The expression of TLR3 (A), TLR7 (B), and TLR8 (C) genes was compared between PBMCs from COVID-19 patients and the control group. Fold changes are displayed to show differences in gene expression across groups. Statistically significant variations (*p* < 0.05) were observed.

According to our findings, COVID-19 patients had significantly higher levels of TLR3 (*p* < 0.001), TLR7 (*p* < 0.001), and TLR8 (*p* < 0.001) gene expression than the healthy control group (Fig. 1).

**Fig. 2:**
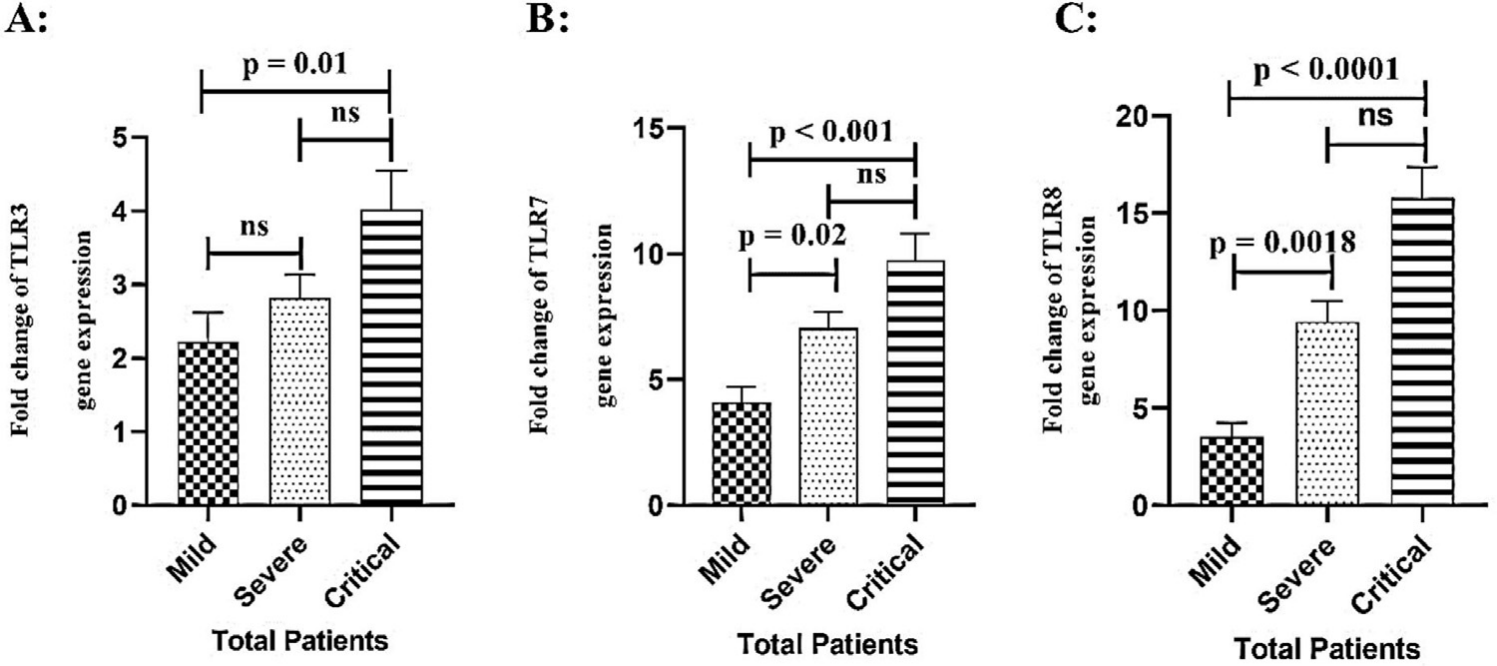
TLR3 A), TLR7 B), and TLR8 C) gene expression was compared in COVID-19 patients during different stages. Fold changes are used to display data. Significant differences across groups (*p* < 0.05).

The results also showed that TLR3 gene expression was significantly higher in the critical COVID-19 patients (*p* = 0.01) when compared to the mild group (Fig. 2A). TLR7 transcript expression levels were also found to be significantly higher in the critical (*p* < 0.001) and severe (*p* = 0.02) COVID-19 patients versus the mild group (Fig. 2B). Furthermore, data analysis revealed that TLR8 gene expression was markedly higher in both critical (*p* < 0.0001) and severe (*p* = 0.0018) COVID-19 patients relative to the mild group (Fig. 2C).

**Fig. 3:**
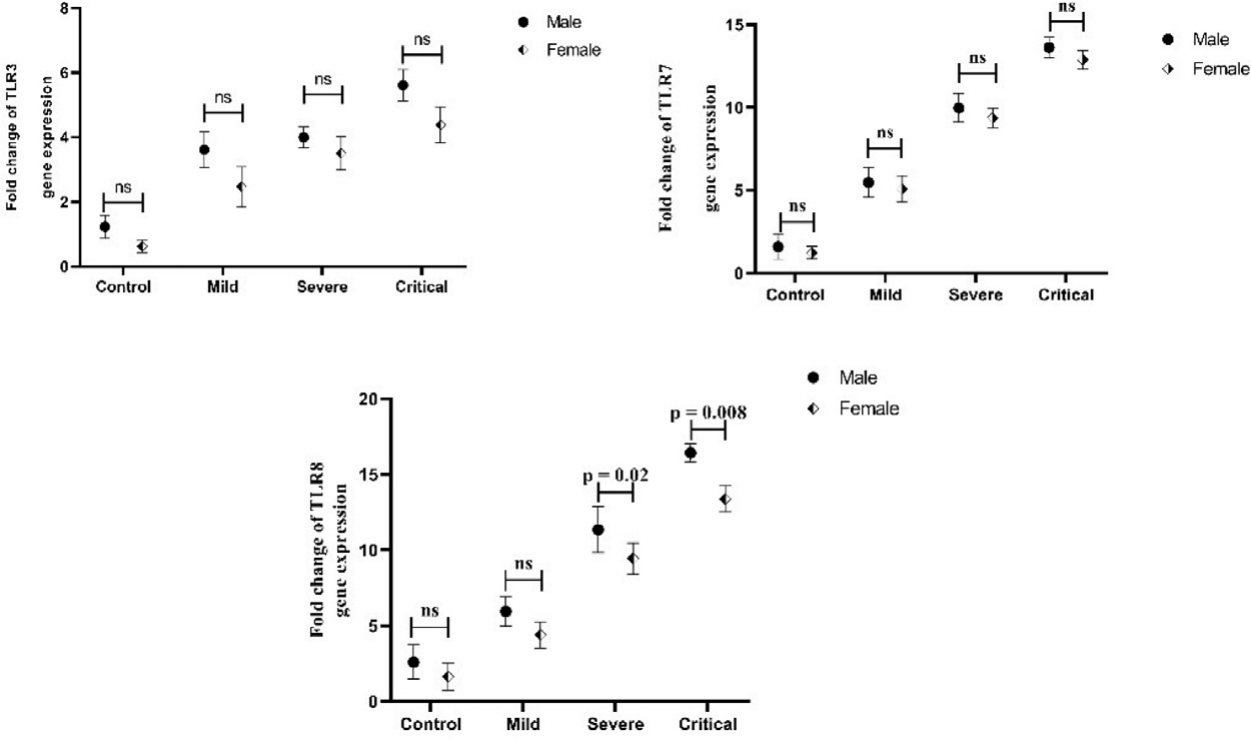
Male and female patients with various illness outcomes were compared in terms of the TLR3 (A), TLR7 (B), and TLR8 (C) genes expression. ns: not significant.

Interestingly, the mRNA levels of TLR8 were significantly higher in male critical (*p* = 0.008) and severe (*p* = 0.02) COVID-19 patients versus female COVID-19 patients at the same stage (Fig. 3C). Our results also showed that as the disease progressed, the expression levels of TLR3 and TLR7 mRNAs in COVID-19 at different stages tended to be higher in male patients than females, but the differences were not statistically significant (Fig. 3A and B).

**Fig. 4:**
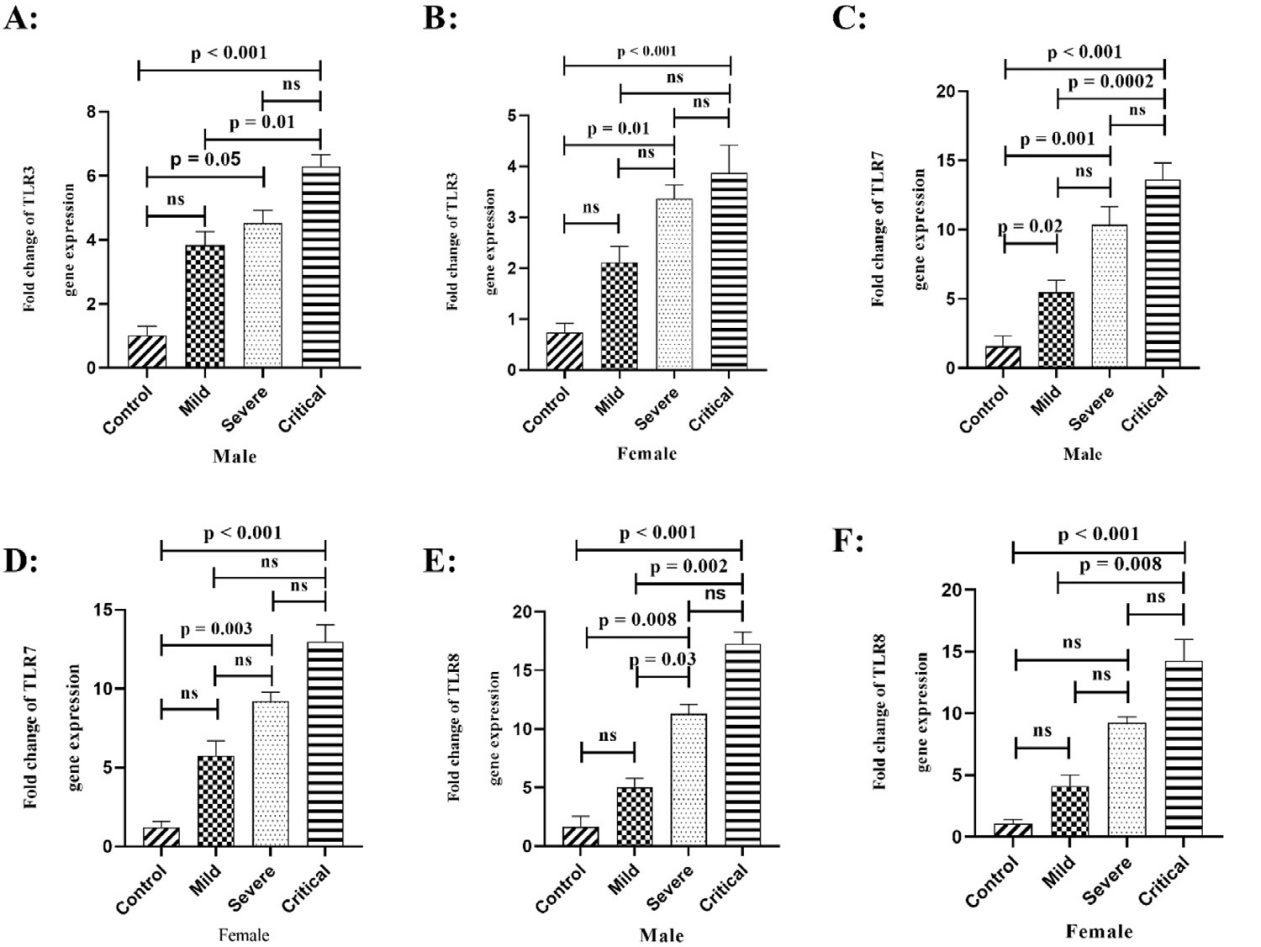
The mRNA expression levels of TLR3, TLR7, and TLR8 were analysed in male and female COVID-19 patients based on disease outcome and gender. Fig. 4A shows TLR3 expression in male patients. Fig. 4B shows TLR3 in females. Fig. 4C depicts TLR7 levels in males while Fig. 4D shows TLR7 in females. TLR8 expression in males is presented in Fig. 4E and in females in Fig. 4F. Data is presented as fold change compared to control. ``ns'' denotes non-significant differences while ``*p* < 0.05'' indicates significant differences between specified groups.

The expression levels of TLR3, TLR7 and TLR8 genes were compared between male and female COVID-19 patients at different stages of disease and healthy control groups. For TLR3, mRNA levels were significantly higher in male critical patients versus mild (*p* = 0.01) and control (*p* < 0.001) groups. Male severe patients also had significantly higher TLR3 expression than controls (*p* = 0.05). Meanwhile, female critical and severe patients showed significantly higher TLR3 versus controls (*p* < 0.001 and *p* = 0.01).

Regarding TLR7, mRNA levels were significantly elevated in male critical, severe and mild groups versus controls (*p* < 0.001, *p* = 0.001, *p* = 0.02). Expression was also higher in male critical versus mild patients (*p* = 0.0002). Female critical and severe patients had significantly higher TLR7 versus controls (*p* < 0.001, *p* = 0.003).

Finally, TLR8 expression was markedly increased in male critical and severe patients versus controls (*p* < 0.001, *p* = 0.008), as well as male critical versus mild patients (*p* = 0.002, *p* = 0.03). Female critical patients showed significantly elevated TLR8 versus mild and controls (*p* = 0.008, *p* < 0.001).

## 4 Experimental Design, Materials and Methods

### 4.1 Experimental design

This case-control study included a total of 100 subjects divided into 4 equal groups: mild COVID-19 patients, severe COVID-19 patients, critical COVID-19 patients, and healthy controls matched for age and gender.

### 4.2 Subjects

75 COVID-19 positive patients were sequentially enrolled from hospitals connected to Kerman University of Medical Science based on clinical criteria for mild, severe and critical disease as defined by WHO guidelines [20]. An additional 25 healthy volunteers matched for age and gender with no underlying conditions served as controls.

### 4.3 Sample collection

6 mL whole blood was collected from all subjects in vacutainer tubes with heparin anticoagulant. Peripheral blood mononuclear cells (PBMCs) were isolated using density gradient centrifugation with Ficoll solution. Cells were counted with a hemocytometer and 1.8 million cells from each sample were stored at –70 °C until use.

### 4.4 RNA extraction and cDNA synthesis

To examine the expression levels of the TLRs, RNA was isolated from PBMCs using a commercially available RNA extraction kit (Favorgen, Taiwan, FAPDE 300) according to the company's instructions. The concentration and purity of the extracted RNA was evaluated by measuring the optical density at 280 and 260 nm wavelengths using a Nanodrop spectrophotometer (Thermo Scientific, USA, ND-1000 UV–Vis). The isolated RNA samples were then reverse transcribed to cDNA using a cDNA synthesis kit (Yekta-Tajhiz, Iran, 201905) according to the manufacturer's protocol. TLR3, TLR7, and TLR8 gene expression was measured using a qRT-PCR system (QIAGEN Systems, Germany, R0510176) and a SYBR green master mix (Amplicon, United Kingdom, A323402). qRT-PCR amplifications with efficiencies ranging from 96 to 106% were performed in triplicate using a 10 µL volume comprising 5 µL SYBR green master solution, 1 µL of the fitting cDNA, and 10 pmol primer and with the temperature conditions of Table 2. The specificity of qRT-PCR was validated by analysis of the melt curve. The expression levels of TLR3, TLR7, and TLR8 were determined using the pfaffl method, and the beta actin gene served as an internal control.

**Table 2:** The sequences of primers, annealing temperature, and real-time PCR conditions.

Gene name	Sequence (5′ -3′)	Annealing temperature (°C)	PCR condition
TLR3	F: 5′- GCTGCAGTCAGCAACTTCAT -3′	61.5	95°C, 5min, 1X
			95°C, 1min 61.5°C, 30 sec 35X 72°C, 1min
	R: 5′- AGGAAAGGCTAGCAGTCATCC -3′		
			72°C, 5min, 1X
TLR7	F: 5′- CTTTGGACCTCAGCCACAACCA -3′	60.1	95°C, 5min, 1X
			95°C, 1min 60.1°C, 30 sec 35X 72°C, 1min
	R: 5′- CGCAACTGGAAGGCATCTTGTAG -3′		
			72°C, 5min, 1X
TLR8	F: 5′- ACTCCAGCAGTTTCCTCGTCTC -3′	58.5	95°C, 5min, 1X
			95°C, 1min 58.5°C, 30 sec 35X 72°C, 1min
	R: 5′- AAAGCCAGAGGGTAGGTGGGAA -3′		
			72°C, 5min, 1X
Beta actin (Endogenous control)	F: 5′- GCACCACACCTTCTACAATG -3′	59.6	95°C, 5min, 1X
			95°C, 1min 59.6°C, 30 sec 35X 72°C, 1min
	R: 5′- TGCTTGCTGATCCACATCTG- -3′		
			72°C, 5min, 1X

### 4.5 qRT-PCR

TLR3, TLR7, TLR8 and beta-actin gene expression was analyzed by quantitative RT-PCR using SYBR Green chemistry on a Rotor-Gene Q instrument. Thermocycling conditions are listed in Table 1. TLR3, TLR7 and TLR8 mRNA levels were quantified by the Pfaffl method using beta-actin as internal reference gene.

### 4.6 Data analysis

Statistical analysis was conducting using SPSS v26 and Graphpad Prism v9. Significance was determined by t-test, ANOVA, and nonparametric tests with *p* < 0.05. Further details are provided in the manuscript text.

## Limitations

• The sample size of 75 COVID-19 patients and 25 controls, while divided into subgroups, is relatively small. Larger cohort studies could provide more statistical power.
• Only TLR3, TLR7 and TLR8 gene expression was examined. Additional innate immune genes and pathways merit investigation as biomarkers.

## Ethics Statement

This study was approved by the Ethics Committee of Shiraz University of Medical Sciences (ethics number IR.SUMS.REC.1402.020, contract number 94.621, clinical trial registration number CRN 1400/21X). All subjects provided written informed consent prior to enrollment. Parental consent was obtained for any participants under 18 years old. The study was carried out in accordance with the Declaration of Helsinki.

## CRediT authorship contribution statement

**Nasir Arefinia:** Methodology, Investigation, Writing – original draft. **Parsa Banafi:** Formal analysis, Validation, Visualization, Writing – original draft. **Mohammad Amin Zarezadeh:** Data curation, Formal analysis, Writing – original draft. **Hawra Shah Mousawi:** Software, Supervision, Validation, Writing – original draft. **Ramin Yaghobi:** Conceptualization, Data curation, Writing – review & editing. **Mehrdad Farokhnia:** Project administration, Resources, Writing – original draft. **Jamal Sarvari:** Conceptualization, Funding acquisition, Writing – review & editing.

## Data Availability

TLR3, TLR7, and TLR8 genes expression in COVID-19 patients (Original data) (Mendeley Data).
